# The calculation of information and organismal complexity

**DOI:** 10.1186/1745-6150-5-59

**Published:** 2010-10-12

**Authors:** Yun Jiang, Cunshuan Xu

**Affiliations:** 1 Key Laboratory of Bioengineering, Henan Normal University, Henan, China

## Abstract

**Background:**

It is difficult to measure precisely the phenotypic complexity of living organisms. Here we propose a method to calculate the minimal amount of genomic information needed to construct organism (effective information) as a measure of organismal complexity, by using permutation and combination formulas and Shannon's information concept.

**Results:**

The results demonstrate that the calculated information correlates quite well with the intuitive organismal phenotypic complexity defined by traditional taxonomy and evolutionary theory. From viruses to human beings, the effective information gradually increases, from thousands of bits to hundreds of millions of bits. The simpler the organism is, the less the information; the more complex the organism, the more the information. About 13% of human genome is estimated as effective information or functional sequence.

**Conclusions:**

The effective information can be used as a quantitative measure of phenotypic complexity of living organisms and also as an estimate of functional fraction of genome.

**Reviewers:**

This article was reviewed by Dr. Lavanya Kannan (nominated by Dr. Arcady Mushegian), Dr. Chao Chen, and Dr. ED Rietman (nominated by Dr. Marc Vidal).

## Background

Organismal complexity is difficult to define and to measure, especially quantitatively. When DNA was discovered to be the material basis of inheritance in all organisms, it was thought that the DNA content of an organism should correlate with its phenotypic complexity, but soon thereafter the C-value paradox was found. C-value refers to the amount of DNA contained within a haploid nucleus, and usually equals to genome size. Salamanders and lungfishes have the largest genomes of 120pg, while the C-value of humans is only 3.5pg [1]. C-values vary enormously among species. In animals they range more than 3,300-fold. Variation in C-values bears no relationship to the complexity of the organism. The discovery of non-coding DNA in the early 1970 s resolved the C-value paradox. Although it is still unclear why some species have a remarkably higher amount of non-coding sequences than others of the same level of complexity, it was believed that the number of genes contained in the genome, rather than the genome size, correlated with the complexity of the organism. However, the human genome project and other model organism genome projects revealed that there are only about 25,000 genes in the human genome [2], while simple organism nematode have 19,500 genes [3] and rice even has more genes than humans, 46,000~55,000 [4]. Obviously, the number of genes bears no direct relationship to phenotypic complexity. This is called G-value paradox.

As proteins are the ultimate bearers of organismal structure and function, it is now believed that the diversity of proteins as well as the interactions between the proteins correlates with the phenotypic complexity. The study of proteomics proceeds extensively after the genome projects. However, proteins are different from nucleic acids. Many proteins are subjected to a wide variety of chemical modifications after translation. A lot of these post-translational modifications, such as phosphorylation, ubiquitination, methylation, acetylation, glycosylation, oxidation, nitrosylation and protein splicing, are critical to the protein functions. The modified proteins display different physical and chemical properties and biological functions from the unmodified. If a modified protein can be seen as a new protein, then the size of the proteome would be much larger than the number of genes. The number of proteins in a living organism may never be accurately counted as the number of genes, because every modified protein can only be individually studied. That is technically much more difficult because different proteins have different properties. A proteomic study can become quite complex even if the object of the study is very restricted. Therefore, it seems difficult to calculate the phenotypic complexity of an organism at least at the present time.

There are some studies about the problem of biological complexity. The traditional mathematical notion of complexity is Kolmogorov complexity, which can be thought of as the length of the shortest message in which the given sequence can be encoded [5]. The complexity is minimal for a homopolymer, and is maximal for a random sequence, in which case complexity is equal to the sequence length. The later case means the sequence cannot be simplified at all, so is the most complex. However, Kolmogorov complexity does not correspond to our intuitive notion of biological complexity. The other complexity is to calculate the Shannon's information entropy of a sequence [5-8]. The complexity is the length of the sequence subtracting the Shannon's entropy, and is actually the information content of the sequence. This kind of complexity has biological meaning. The more complex means the sequence is more conserved and therefore carries more information. However, this complexity is only the complexity of sequences, which has nothing to do with the phenotypic complexity of organisms.

There are three parts of information contained in an organism's genome: first, the information to construct the organism; secondly, the information to constitute DNA structures, including replicons, centromeres, telomeres, etc; and finally, the information for the mechanisms of evolution, which we do not know at the present time. Here we take the amount of minimal information needed to construct an organism as a measure of organism phenotypic complexity because apparently more complex organisms should need more minimal information to construct and simple organisms are supposed to need less information to construct. The information to constitute DNA structure is relatively simple and less important, and we know very little about the mechanisms of evolution, so for the purpose of this article we will not be considering these areas of information. The information needed to construct organism, to put it simply, is the information needed to express proteins in time, in space, and in quantity. We actually still know very little about this information up to now. The conserved gene coding sequences are only part of the information. To calculate this information, we need to construct organism mathematically using permutation and combination formulas based on the numbers of proteins and cell types.

## Results

### The calculation of information

While some biologists may know how to calculate the information content of sequence, most are not familiar with how to calculate amount of information. According to Shannon's information concept, information is to decrease uncertainty. The more uncertainty information decreases, the more the information. Information is the difference between the entropy of known and unknown. How much uncertainty you need to decrease, how much information you need. If you know everything, you do not need information. The less you know, the more you need to know, the more information you need. The uncertainty can often be calculated as possibility. The certainty means only one possibility. The uncertainty means many possibilities. The more possibilities excluded, the more the information.

Shannon's information entropy can be calculated using formula:

$$H=-\Sigma p\cdot \log_{2}(p)$$

where p are the probabilities of events.

When the probabilities of all events are equal, *H *gets the maximal value. Let *N *be the number of events or possibilities, then

$$H=-\sum \frac{1}{N}\cdot \log_{2}(\frac{1}{N})=\log_{2}(N)$$

The number of possibilities *N *can be calculated by using permutation and combination formulas. *H *is the entropy of unknown because you only know the probabilities or possibilities. When you know everything, *H *becomes 0 because there is only one possibility left with probability 1 and the probabilities of other possibilities are 0. The entropy of known is 0. In order to know, you need to reduce the entropy to 0, so you need information:

$$I=H_{\text{unknown}}-H_{\text{known}}=H-0=H=\log_{2}(N)$$

So information *I *has the same value as entropy *H*, but they are different concepts. Entropy is a quantity to describe disorder. Information is a quantity to reduce disorder. Information can be calculated based on the entropy you need to reduce. It is easy to calculate information if the number of possibilities can be calculated. For example, in order to guess a random 8-digits telephone number, you need information

$$I=\log_{2}(10^{8})=26.57\text{ bits}$$

The probability for each event is same here. Sometimes the probability for each event may not be same, but you do not know the probabilities, so any probability distribution is possible and equal probability distribution is also possible. In order to know, you have to need information to reduce the entropy from the maximum to 0. You have to assume equal probability because in this way you need minimal information. For example, we need to encode a protein sequence with 10 amino acids (all the amino acids are independent). Although the actual amino acids distribution across the sequence may not be equal, we do not know the distribution information. We have to assume equal probability to write the sequence. In this way, we need minimal information. The formula *I=*log*(N*) can still be used. We need information

I=10⋅log(20)=43.2 bits

to write a protein sequence with 10 amino acids. If we know the sequence is MMMMMMMMMM, we need information *I*=0 to write the sequence because it is already known. If we know the sequence is a DNA sequence, then the entropy of unknown is *H = *10 log(4), so we need information

I=10⋅log(4)=20 bits

to write the sequence. If we know it is an English sequence but we do not know any English except 26 letters, then the entropy of unknown is *H*=10 log(26). We need information

I=10⋅log(26)=47 bits

to write the sequence. If we know English quite well, we will need much less information to write the sequence. For example, for the last character of the sentence "I love yo_", you may need no information to guess the underscore is "u" if you know English, but I need information log(26) bits to guess the character if I do not know English except 26 letters. Even though the distribution of each English letter is actually not equal at all, but I do not know the probabilities of each letter, I have to assume equal probability. I still need information log(26) bits for each character. Different persons need different information to know because they know differently. Information is the difference between the entropy of known and unknown. How much information you need depends on how much you have known, therefore depends on the entropy of unknown. The more you know, the less the entropy of unknown, and the less the information you need. For a sequence, we can also say the information of the sequence is the information we need to write the sequence.

Another example is to calculate the regulatory information of viruses. If a virus has 10 protein coding genes and all the genes express one by one in sequence and only express once, how much should be the regulatory information? The probability for each gene expression is the same, so the formula *I=*log(*N*) can be used. For the first gene to express, you have 10 genes to choose. For the second gene, the number of genes to choose is 9. For the third gene, you have 8 genes to choose... For the last gene, only one gene left. So the number of possibilities for all genes expression is:

N=10⋅9⋅8⋅⋅⋅2⋅1=10!=3,628,800

The information needed for the order of expression is:

I=log(N)=log(10!)=21.8 bits

This information is regulatory information, which is actually composed of regulatory sequences or Transcription Factor (TF) binding sites. Because every base pair of DNA contains information log(4) = 2 bits, the length of all TF binding sites should be at least 21.8/2 = 10.9 bp. A virus may use longer sequence for the binding sites, but the minimum should be 11 bp.

The concept of information content of sequence is a little different from the concept of information above. The calculation of information content also needs to use Shannon's information entropy. To calculate information content, you must know the probability distribution of each site of a sequence, which is based on the data of population genetics. The information content or complexity of the sequence can be calculated as [5,7,8]:

$$C=H_{\max}-H_{\text{known}}=L-H$$

where *L *is the length of the sequence, *H *is the entropy of known. For example, for a sequence AXT, the first site A is very conserved with probability 1 for A, 0 for other base pairs. The information content of the first site is:

$$C_{1}=1-\log_{4}(1)=1-0=1\;\text{or}\;C_{1}=\log_{2}(4)-\log_{2}(1)=2-0=2\text{ bits}$$

If the second site X is actually random, any bases are possible, the information content is:

*C*_2 _= 1-1/4·log(4)·4 = 1-1 = 0 or *C*_2 _= log_2_(4)-1/4·log_2_(4)·4 = 2-2 = 0bit

If the probabilities of the third site T are 1/2 for T, 1/4 for A, 1/4 for G, the information content of third site can be calculated as:

$$\begin{array}{l} C_{3}=1-[\frac{1}{2}\cdot \log_{4}(2)+\frac{1}{4}\cdot \log_{4}(4)+\frac{1}{4}\cdot \log_{4}(4)]=1-\frac{3}{4}=\frac{1}{4}\\ or\\ C_{3}=\log_{2}(4)-[\frac{1}{2}\cdot \log_{2}(2)+\frac{1}{4}\cdot \log_{2}(4)+\frac{1}{4}\cdot \log_{2}(4)]=2-1.5=0.5bit \end{array}$$

So the information content of this sequence is:

C=1+0+1/4=1.25 or C=2+0+0.5=2.5 bits

The more conserved the sequence, the more the information content. The amount of information of the sequence is determined by how much you know to write the sequence. If you know it is a DNA sequence and the probability distribution of each site of the known sequence, you need information *I = H*_unknown_-*H*_known _= 6-3.5 = 2.5 bits to write the sequence AXT. The information of the sequence is 2.5 bits.

For each genetic codon, which is composed of 3 base pairs, the amount of information is *I*=*H*_unknown_- *H*_known _=3 log_2_(4)-0 = 6 bits. For each amino acid, because many amino acids have more than one codon, the information content is different. For example, proline has 4 codons and the third base pair of the codons is wobbled, the information content of proline *C=H*_max_-*H*_known_=6-2 = 4 bits. In the same way, the information content of Arg is 3 bits, of Asn is 5 bits, and of Trp is 6 bits. Organisms probably know the importance of each amino acid and how to regulate gene expressions with codon bias, and evolved this codon system to regulate translation efficiently and reduce the harm of mutation of important amino acids. The redundance of information is *R=I-C*. The less the information content is, the more the redundance of information, the more the space to silence or neutralize mutation. So Arg is more important, more protected from mutation, and more expression regulated than Trp. Although some amino acids may need less information to be encoded (for example, 2 base pairs for the amino acids with information content less than 4 bits), organisms actually use 3 base pairs for every amino acid, so it cost organisms 6 bits genomic information to encode every amino acid. In another word, the amount of information of every amino acid in genome is 6 bits although there is information redundance in the 6 bits.

Using fixed number of base pairs rather than different number to encode all amino acids manifests that organisms probably do not know the distribution of amino acids across all protein sequences and assume equal probability for all amino acids. In this way organisms can change protein sequences and amino acid distributions flexibly and efficiently under changing environments. Otherwise, organisms have to change the codon system when the distributions change. For evolution, it is impossible for a living organism to know the future protein sequences and the amino acid distributions across the sequences. In order to generate any unknown future sequences efficiently, living organisms need to use a mechanism that can encode any protein sequences efficiently. In fact, all organisms use a mechanism that can reduce the maximal entropy to 0, which means equal probability for all amino acids when encoding protein sequences. For the same reason, organisms may not know the distribution of bases across all nucleotide sequences, and may assume equal probability for all bases even though the actual probability distribution across some genomes may not be equal at all. For biological information, most cases are like this with maximum entropy needed to be reduced and equal probability need to be assumed by organisms, so formula *I=*log(*N*) can be used to calculate the information.

Fixed number of base pairs of genetic code also simplifies the reading of DNA protein coding sequence and reduces the total information needed to encode protein. If the lengths of codons were different, "space" would have to be used to separate each codon in protein coding sequence, and the total mechanisms of translation would be more complex.

### The calculation of effective information of viruses

Although proteins and other molecules may contain information beside DNA genetic information, most information that can pass to next generation is in DNA, so DNA directly controls enough information to construct organism. All the processes not directly controlled by DNA, including post-translational protein modifications and interactions, are the functions of proteins and will proceed automatically when the proteins are synthesized. All the information necessary for these functions is already contained in DNA sequences. Therefore, we do not need to take the post-translational processes into consideration when calculating genomic information needed to construct an organism.

The term "effective information" can be used to describe the minimal amount of information needed to construct an organism. The effective information actually means the genome size except "junk DNA" or "functional fraction" of genome. There are different formulas to calculate the effective information of viruses, bacteria, and eukaryotes because they have different genomic information structures.

Every organism has proteins, which need to be expressed in order, in time, and in quantity. Viruses have several to hundreds of proteins. For a complex virus with hundreds of proteins, it may need to separate expression of early and late proteins and maintain precise ratios of different protein products expressed simultaneously. Because all virus proteins are expressed only once, we only need to consider the order of virus protein expressions, while the quantities of protein expressions are controlled by the feedback through the affinity of regulatory factors to the binding sites.

There are many possibilities for the order of virus protein expressions and the protein sequences. If each protein is expressed one by one, the number of possibilities *N *can be calculated by using permutation and combination formula:

$$N=x!\cdot 20^{n_{1}}\cdot 20^{n_{2}}\cdot \cdot \cdot 20^{n_{x}}$$

where *x *is the number of proteins, *n*_1_, *n*_2_, ... *n*_x _are the length of protein sequences (the unit is the number of amino acid residues).

As the probabilities for all amino acids and proteins are equal as analyzed before, the effective information can be calculated:

I=log(N)=log(x!)+Σn⋅log(20)=log(x!)+Σn⋅4.32

where n are lengths of virus proteins.

The protein expressions may not be one by one in sequence. Some proteins may be expressed simultaneously. In this way, organisms may use less information to express proteins, depending on the specific expression routes. However, this way will be inflexible to change the routes under changing environments. Like encoding protein sequence, organisms may possibly use a mechanism that can cope with any routes and reduce the maximum entropy to 0. The maximum entropy is the case that all proteins are expressed one by one in sequence. In this way, organisms can change the expression routes easily without changing the mechanism of how the information system works.

Genetic information is stored in the form of DNA. DNA genetic code has degeneracy. In order to better compare the value of *I *and the genome size, we will not calculate the actual minimal information but the DNA information. So the formula becomes:

$$I=\log(x!)+\Sigma n\cdot 3\cdot \log_{2}(4)=\log(x!)+\Sigma n\cdot 6=I_{2}+I_{1}$$

where 3 is the number of base pairs that make up a codon. The information *I*_1 _forΣ*n *6 is of the protein coding sequences, because it is just the length of protein coding genes in bits. The information *I*_2 _for log(*x*!) is of regulatory sequences. The *x *should be the number of protein linkage groups or operons. If all virus proteins are in one operon or linkage group or expressed simultaneously, no regulatory information is necessary. Because the number of protein linkage groups is usually unknown, we still use the number of proteins instead. The real *I*_2 _in viruses is very possibly equal to *x *log(*x*) because in this way viruses can use fixed length of regulatory sequence log(*x*) for every gene or operon. This may simplify the mechanism of regulation. Although the information *I*_2 _is calculated by assuming one by one protein expression, the result is that each gene or operon has a regulatory sequence, which is very possibly true.

The amount of effective information of *Avian infectious bronchitis virus *is: *I*=76,512 bits. The genome size is: *C*=length of genome 2 = 55,216 bits. So the *I *value is larger than the C value. After checking the virus genome, we find there is an overlap between genes. It's the overlap that makes the *I *value too large. When calculating the information of the overlapping part of the genes for the second time, because these base pairs already cannot change any more and there are no possibilities to be excluded, the amount of information for the second time calculation is 0. For example, sequences AACCC and CCCGG have overlapping part CCC. The information of the first sequence is 5 2 = 10 bits. The information of the second sequence is 2 2 = 4 bits because the information of sequence CCC is already known and this part contains no new information. So the information of sequence AACCCGG is 14 bits rather than 20 bits. The actual value of *I*_1 _should be exactly equal to the size of the protein coding area. For virus genomes with overlapping genes, we use the actual size of protein coding area instead of Σ*n *6 to calculate the effective information.

The effective information of some viruses is as follows (Table 1).

**Table 1:** The effective information of viruses (the numbers in parentheses are the numbers of proteins)

Virus name	*I *(bit)	C (bit)
*African cassava mosaic virus*(ssDNA)(9)	8,233	11,042
*Australian bat lyssavirus *(ssRNA)(5)	21,523	23,644
*Avian infectious bronchitis virus *(ssRNA)(6)	52,465	55,216
*Barley yellow dwarf virus-PAV *(ssRNA)(7)	9,777	11,354
*Bat coronavirus Rp3/2004 *(ssRNA)(13)	57,720	59,472
*Beak and feather disease virus *(ssDNA)(3)	3,271	3,986
*Bean common mosaic necrosis virus *(ssRNA)(1)	18,396	19,224
*Bean golden yellow mosaic virus *(ssDNA)(8)	7,878	10,464
*Beet curly top virus *(ssDNA)(6)	5,278	5,988
*Beet mosaic virus *(ssRNA)(1)	18,510	19,182
*BK polyomavirus *(dsDNA)(6)	9,182	10,306
*Bovine coronavirus *(ssRNA)(12)	60,223	62,056
*Bovine polyomavirus *(dsDNA)(6)	8,558	9,394
*Cabbage leaf curl virus *(ssDNA)(7)	7,875	10,192
*Canary circovirus*(ssDNA)(2)	3,240	3,904
*Cestrum yellow leaf curling virus *(Retro-transcribing)(7)	14,372	16,506
*Human herpesvirus 1 *(dsDNA)(77)	240,948	304,522
*Human SARS coronavirus *(ssRNA)(14)	56,411	59,502
*Murine polyomavirus *(dsDNA)(6)	9,544	10,594
*Porcine epidemic diarrhea virus *(ssRNA)(6)	54,393	56,066
*Vaccinia virus *(dsDNA)(223)	344,114	389,422
*Zucchini yellow mosaic virus *(ssRNA)(1)	18,480	19,182

The effective information *I *of viruses range from thousands of bits to hundreds of thousands of bits. In general, single strand DNA viruses have the minimum amount of effective information, down to 3 thousands bits, while double strand DNA viruses have the maximum amount of effective information, up to hundreds of thousands of bits. Single strand RNA viruses and retro-transcribing viruses are between the above two.

The amounts of calculated effective information of viruses are consistent to the complexity defined by the number of proteins or genome size. In fact, the effective information is roughly proportional to the number of proteins. For viruses, the advantage of using effective information is not obvious because viruses are simple and the number of protein coding genes or genome size can be used to determine the complexity of viruses. For higher multicellular organisms, due to G-value paradox, the effective information will be more useful.

### The calculation of effective information of Bacteria

The calculation of effective information of bacteria is more complex than viruses' because the genes may express more than once in the growth of bacteria. There are regulations on protein sequency and quantity, but no regulation on the spatial deployment. The proteins can be thought to reach their positions in a cell automatically after synthesized.

A grown bacterium has roughly fixed volume, mass, and the number of protein molecules. It needs to produce all the protein molecules in its growth. The production of proteins in the growth is like chain reactions controlled by complex regulatory cascades with feedbacks. Even though the production of proteins is complex, we can always think that a bacterium grows theoretically from producing the first protein molecule to the last molecule in sequence. In this way, we can calculate the effective information. The problem is that many molecules may be produced simultaneously in the process. This may need less information. However, like viruses, in order to change protein expressions flexibly, organisms may use an information mechanism that can produce protein molecules one by one in sequence to reduce the maximum entropy to 0, which means each protein or operon has at least one regulatory sequence. Even if the calculated information may be more than necessary, we can still use *k *value later to adjust the information to fit to the actual amount of effective information based on real regulatory information of organisms.

The number of possibilities for the first molecule is:

$$N_{1}=x\cdot 20^{n_{1}}$$

where *x *is the number of proteins, *n*_1 _is the length of the first protein.

If all the molecules are independent each other, the number of possibilities for all molecules by using permutation and combination formula is:

$$N=x^{y}\cdot 20^{n_{1}}\cdot 20^{n_{2}}\cdot \cdot \cdot 20^{n_{x}}$$

where *y *is the number of all protein molecules in a grown bacterium, *n*_1_, *n*_2_, ... *n*_x _are the length of proteins.

The regulatory information is calculated as encoding the order of all protein molecules in a cell, which includes timing and volume of expression. If you know the first 10 molecules produced are protein A, the next 100 molecules are protein B, the next 50 molecules are protein A again, the last 200 molecules are protein Z, you know the timing and volume of expression.

Although the chance for each protein in an organism may not be equal, the distribution is actually unknown for the organism because the distribution always needs to change under changing environment. For similar reason for encoding protein sequences, in order to cope with changing environments, organisms have to use a mechanism that can generate any distribution, including equal distribution, to reduce the maximal entropy to 0. This kind of mechanism provides flexibility for organisms to change the quantities of certain proteins any time to adapt the environments.

The effective information can be calculated:

$$I=\log(N)=y\cdot \log(x)+\Sigma n\cdot \log(20)=I_{2}+I_{1}$$

The effective information includes two parts: first, the information to encode the protein sequences (*I*_1_), and secondly, the regulatory information (*I*_2_). The formula to calculate *I*_1 _is the same as virus's. Converting the information to DNA information, the calculation can be simplified as:

$$I_{1}=\Sigma n\cdot 6=6nx$$

where *n *is the average length of bacteria proteins, which is 308 for all bacteria[9]. *I*_1 _should be adjusted to the exact size of the proteins coding area if there is overlapping of genes.

For regulatory information *I*_2_, in fact, every protein molecule is not produced independently. A bacterium does not once synthesize only one protein molecule, but a batch of protein molecules. Only the first protein molecule in the batch is independent, the possibility is × kinds of protein. The other following protein molecules are not independent. The possibilities follow the previous protein, which can only be 1. If a bacterium synthesizes 100 protein molecules at a time, *I*_2 _is:

$$I_{2}=y/100\cdot \log(x)$$

It can be expected that the number of protein molecules a bacterium once synthesizes may be proportional to the average quantity of the proteins *y/x*, which means the bigger the bacterium of the same structural complexity, the more the protein molecules it synthesizes at once.

$$I_{2}=k\cdot y/(y/x)\cdot \log(x)=k\cdot x\cdot \log(x)=kx\log(x)$$

where *k *is the average number of times a protein is synthesized. The value of *k *can be estimated as 5 based on the genomic information structure of *E. coli *(about 3% of E. coli genome is estimated as regulatory information). This is equivalent to that *E. coli *synthesizes 67 same protein molecules on average at a time. When the bacteria are very small, *y *is very close to *x*. The quantity of a kind of protein molecules may be less than 5. In order to ensure at least one protein molecule synthesized at once, the formula needs to be calibrated as:

$$I_{2}=\frac{kx}{1+k\cdot x/y}\log(x)$$

This formula means that each protein has at least one regulatory sequence. The average number of regulatory sequences is *k*. The quantity of a certain protein is controlled by the times the protein is synthesized. The more the quantity, the more the times the protein is synthesized, the more the regulatory sequences. The average number of times is *k*. The quantity of protein synthesized each time is not directly determined by DNA information, but determined by the feedback through the affinity of regulatory factors to the binding sites. It is the number of regulatory binding sites upstream the protein coding gene sequence that determines the quantity of the protein.

In fact, most bacterial proteins are synthesized within the unit of operons, i.e. all the proteins in an operon are linked. They are not independent and are synthesized together. So the *x *in the formula should be the number of the addition of all operons and independent proteins in the genome. For example, the number of operons in *E. coli *is 834 [10], with the number of independent proteins, together amounts to about 2,584. The *x *should be 2,584 for *E. coli*. Because the number of operons in most bacteria genomes is still unavailable at the present time, we calculated *I*_2 _based on the number of proteins.

The complete formula to calculate the amounts of information of bacteria is:

$$I=I_{1}+I_{2}=6nx+\frac{5x}{1+5x/y}\log(x)$$

where *y *is the total number of protein molecules in a bacterium. The effective information of some bacteria is as follows (Table 2).

**Table 2:** The amounts of effective information of bacteria

Bacterium name	x	v(μm^3^)	I (bit)	C (bit)	I1(bit)	I2/C
*Acetobacter pasteurianus*	2628	1.00	5.00e6	5.8e6	4.86e6	2.6%
*Acinetobacter baumannii*	3351	1.15	6.39e6	7.8e6	6.19e6	2.5%
*Bacillus anthracis*	5300	13.27	1.01e7	1.08e7	9.79e6	3.0%
*Bacillus subtilis*	4176	1.51	7.97e6	8.4e6	7.72e6	2.9%
*Bordetella pertussis*	3436	0.0082	6.47e6	8.1e6	6.35e6	1.4%
*Clostridium botulinum*	3425	4.02	6.53e6	7.7e6	6.33e6	2.6%
*Clostridium perfringens*	3301	14.13	6.29e6	7.8e6	6.10e6	2.5%
*Clostridium tetani*	2373	2.26	4.52e6	5.6e6	4.39e6	2.3%
*Corynebacterium diphtheriae*	2272	0.523	4.32e6	4.97e6	4.20e6	2.5%
*Coxiella burnetii*	1866	0.065	3.54e6	4.01e6	3.45e6	2.4%
*Escherichia coli*	5105	0.588	9.74e6	1.15e7	9.43e6	2.7%
*Francisella tularensis subsp*.						
*Holarctica*	1521	0.0042	2.86e6	3.7e6	2.81e6	1.3%
*Haemophilus influenzae*	2307	0.1413	4.39e6	3.7e6	4.26e6	3.4%
*Klebsiella pneumoniae*	4776	0.9813	9.12e6	1.06e7	8.83e6	2.8%
*Lactobacillus delbrueckii subsp*.						
*Bulgaricus*	1715	4.52	3.26e6	3.7e6	3.17e6	2.5%
*Legionella pneumophila*	2942	0.113	5.60e6	6.8e6	5.44e6	2.4%
*Mycobacterium avium*	4634	0.9043	8.84e6	9.7e6	8.56e6	2.9%
*Mycobacterium tuberculosis*	3596	0.785	6.86e6	8.7e6	6.65e6	2.4%
*Mycoplasma pneumoniae*	689	0.00818	1.30e6	1.6e6	1.27e6	1.8%
*Neisseria meningitidis serogroup A*	1909	0.268	3.63e6	4.37e6	3.53e6	2.3%
*Pseudomonas aeruginosa*	5892	0.097	1.12e7	1.30e7	1.09e7	2.6%
*Salmonella enteritidis*	4206	1.154	8.02e6	9.3e6	7.77e6	2.7%
*Salmonella typhimurium*	4423	0.2943	8.43e6	9.7e6	8.17e6	2.7%
*Shigella dysenteriae serotype 1*	4419	0.848	8.43e6	1.05e7	8.17e6	2.5%
*Staphylococcus aureus*	2660	0.5233	5.07e6	5.6e6	4.92e6	2.7%
*Streptococcus pneumoniae*	2386	0.3815	4.54e6	4.1e6	4.41e6	3.2%
*Streptococcus pyogenes serotype M1*	1696	0.5233	3.22e6	3.7e6	3.13e6	2.4%
						

The amounts of effective information of bacteria range from millions of bits to tens of millions of bits, just one order of magnitude higher than viruses'. The regulatory information, except very small bacteria and mycoplasma, accounts for 2~3% of the genome. The *I *values correlate well with the bacterial complexity defined by number of protein-coding genes, genome size, and volume. Bacteria's *I *values are higher than virus's. This is also consistent with our knowledge about complexity.

The only thing that looks like an anomaly is that the *I *value of *Haemophilus influenzae *and *Streptococcus pneumoniae *exceeds the C value. Checking the average length of the proteins, we find that the actual protein average length of *Haemophilus influenzae *is only 235 (< 308) and the actual size of protein coding area is only 3.26e6 bits, while the calculated *I*_1 _value is 4.26e6 bits, which already exceeds the C value. The corrected actual *I *value of *Haemophilus influenzae *is 3.39e6 bits, accounting for 91.6% of C value. Similarly, the average length of proteins of *Streptococcus pneumoniae *is 250, which is also much lower than the average value of bacteria. The corrected actual *I *value of *Streptococcus pneumoniae *is 3.77^e^6 bits, accounting for 92% of C value. Therefore, if precise *I*_1 _value is needed, it should be directly calculated from the actual size of protein coding area, which is: *I*_1 _= C•%coding

### The calculation of effective information of eukaryotes

While the calculation of effective information of unicellular eukaryotes is the same as bacteria's, the calculation of *I *of multi-cellular organisms is much more complex because multicellular organisms not only need to produce all proteins to build different cells, but also need to put all the cells in spatial structures to build the organisms.

The number of possibilities for all the cells put together and the effective information can be calculated by using permutation and combination formula similarly, but the equation is very long and the explanation can be quite complex. It is better to separate the effective information directly to three parts and calculate separately: first, the information to encode all the proteins *(I*_1_); secondly, the information to produce all the differentiated cells (*I*_2_); and finally, the information to construct the spatial structures (*I*_3_).

The information to encode all the proteins is the size of the protein coding area in the genome, like bacteria's *I*_1_. There are also overlapping genes in eukaryotic genomes, and the overlapping genes account for considerable weight [11]. So these overlapping parts must be adjusted according to the actual size of protein coding area, otherwise these parts will mix with other parts of information and can cause confusing results.

If the genome is not yet sequenced, this information can be calculated:

$$I_{1}=6n\cdot g$$

where *g *is the number of protein coding genes, *n *is the average length of proteins of eukaryotes, which is 448 [9]. In this way, the calculated *I*_1 _is usually larger than the actual size of protein coding area.

To produce all the differentiated cells, proteins need to be chosen from the complete proteome. For one type of cells, the algorithm is similar to bacteria's,

$$I_{2}=k_{2}\cdot x\cdot \log(x)=\log(x^{xk_{2}}).$$

Let *x *be the size of complete proteome (number of functional proteins before post-translational modifications), let *t *be the average size of cellular proteome of differentiated cells, *cn *is the number of cell types, *a*_1_, *a*_2_, *a*_3_,..., *a*_cn _are the diversities of differentiated cellular proteomes from *t*. Because many genes expressed in the differentiated cells are the same, *t*+*a*_1_+*a*_2_+*a*_3_+...+*a*_cn_=*x*, then the information to produce diverse differentiated cells is:

$$\begin{array}{l} I_{2}=\log(C_{x}^{t+a_{1}}\cdot t^{(t+a_{1})k_{2}}\cdot C_{x-t-a_{1}}^{a_{2}}\cdot t^{a_{2}k_{2}}\cdot C_{x-t-a_{1}-a_{2}}^{a_{3}}\cdot t^{a_{3}k_{2}}\cdot \cdot \cdot C_{a_{cn}}^{a_{cn}}\cdot t^{a_{cn}k_{2}}\\ =\sum \log(C)+k_{2}\cdot (t+a_{1}+a_{2}+\cdot \cdot \cdot +a_{cn})\cdot \log(t)=\sum \log(C)+k_{2}\cdot x\cdot \log(t) \end{array}$$

where *k*_2 _is a coefficient. The formula means choosing *t+a*_1 _from × to construct the first type of cell, and choosing *a*_2 _from *x-t-a*_1 _to construct the second cell, and so on. The possibilities for every protein molecule in all cell types are *t*, and the numbers of free protein molecules are proportional to the diversities of cellular proteome. As the calculated value of Σlog(C) is actually quite small and can be negligible, the information to produce diverse differentiated cells is:

$$I_{2}=k_{2}\cdot x\cdot \log(t)$$

For unicellular eukaryotes, the calculation of *I*_2 _of is the same as bacteria's:

$$I_{2}=k_{1}\cdot x\cdot \log(x)$$

where *x *is the number of proteins. *k*_1 _is estimated as 30 based on the information structure of *Saccharomyces cerevisiae *(Baker's yeast) genome to make the amount of effective information account for about 80% of the genome. This is equivalent to that a yeast cell synthesizes average 1000 protein molecules at once.

Because there are regulations between the cells of multicellular organisms, *k*_2 _can be larger than *k*_1_, estimated as 110, which is equivalent to a cell synthesizes average 820 protein molecules at a time.

The average number of genes expressed in specific tissues is about 5000. The proteome size of a specific tissue ranges from a few thousand to tens of thousands. Because a tissue contains diverse cells, the proteome size of one differentiated cell may be a few thousand. Because most proteome data are still unavailable and are confusing with the proteome containing post-translational modifications, transcriptome data from clustered EST can be used instead. The number of unique sequences of clustered ESTs from human breast tumors is 6501[12], so we estimated *t*=6500 as the average cellular proteome size of differentiated cells.

$$I_{2}=110\cdot x\cdot \log(6500)=110\cdot \log(6500)x$$

With all the differentiated cells available, organism spatial structure can be constructed. Let *z *be the total number of cells in an organism.

In the process of organismal development, cell divisions are controlled by complex regulatory signals. We can always think that cells are produced from the first one to the last one in sequence. When the last cell is produced, the development ends and the organism reach its adult weight. In this way, information can be calculated. Although some cells may be produced simultaneously, organisms may use an information mechanism to reduce the maximum entropy to 0, which is the case that all cells are produced one by one in sequence, in order to keep the flexibility to change the cell development routes easily. Even if the calculated information may be more than necessary, it can still be adjusted by *k*_3 _value later to fit to the actual amount of information.

Like cell division, there is only one appropriate position between two adjacent cells. In this way, the spatial structure can be easily constructed. Let us start from deploying the first cell. For the first cell, the possibility is *cn*, i.e. there are *cn *kinds of cells to choose. For the second cell, the possibility is *cn *1, i.e., there are *cn *kinds of cells to choose and the cell can only be deployed adjacent to the first cell (divided from the first cell). The possible position is only 1. For the third cell, the possibility is *cn *2, i.e. there are *cn *types of cell to choose and the cell can be divided from the first cell or the second cell. There are 2 positions to choose. When deploying the last cell (the *z*th cell), the possibility is *cn (z*-1), i.e. there are *cn *types of cell to choose and the cell can be divided from any (*z*-1) cells. There are *z*-1 positions to choose.

If all cells are independent, the number of possibilities to produce and deploy all cells can be calculated using permutation and combination formula:

$$N=cn^{z}\cdot (\text{z}-1)!$$

The information is:

$$I_{3}=\log[cn^{z}\cdot (\text{z}-1)!]=\sim z\cdot \log(cn)+\log(z!)$$

The chances for different cell types and different positions may not be equal, but they are unknown. Organisms have to use mechanisms that can generate all possible distributions of cells and positions, including equal distribution. This kind of mechanisms provides flexibility for organisms to change the quantities and positions of cells easily and efficiently to adapt the environments.

When *z *is larger than 10^5^, log*(z*!) is very close to *z*log(*z*) (> 90%). In order to simplify the calculation, log(z!) is replaced by *z*log(*z*). When *z *is small, log(*z*!) is still used.

$$I_{3}=z\log(cn)+z\log(z)=z[\log(cn)+\log(z)]$$

*z *can be calculated from the average adult body weight.

In fact, not all the cells are generated and deployed independently. Usually a batch of cells are generated and deployed at once. Only the first cell of the batch is free. The possibilities of cells afterward can only be 1. If all the cells of one type are averagely generated by one time, then

$$I_{3}=z/(z/cn)\cdot [\log(cn)+\log(z)]=cn\cdot [\log(cn)+\log(z)]$$

The actual situation is that all the same cells are generated and deployed by many times.

$$I_{3}=k_{3}\cdot cn[\log(cn)]+\log(z)]$$

where *k*_3 _is the average number of times one type of cells is generated. *cn *should be the number of cell types or the number of linkage groups. In fact, in the organs or tissues of multicellular organisms, many cells are linked, causing repetitive pattern in the organ or tissue. So the numbers of cell types *cn *in the formula should be replaced by the number of linkage groups. There is no regulatory information needed for genes or cells inside a linkage group. However because the numbers of linkage groups in organisms are still unknown, we calculated *I*_3 _based on the number of cell types.

The formula also means each type of cells has at least one regulatory sequence. The average number is *k*_3_. The quantities of each type of cells are controlled by the number of times this type of cells is generated. The more the quantity, the more the times this type of cells is generated. The quantity of cells generated each time is not directly determined by DNA information, but by the cellular feedback signals. It is the times of generating that determines the quantities of each type of cells.

*k*_3 _can be estimated as 2.5e4, which is equivalent to that human body averagely generates and deploys 4.2e7 cells at once, that is about 0.012g. The *k*_3 _value is determined by the information structure of *Schistosoma mansoni *and *Caenorhabditis elegans*. Because *C. elegans *is very small and the *I*_3 _of which is almost negligible, and *Schistosoma mansoni *is bigger than *C. elegans *but the phenotypic complexity should be a little bit less than *C. elegans*, therefore we adjusted the *k*_3 _value to make the *I *value of *Schistosoma mansoni *close to, but a little bit lower than the value of *C. elegans*. So we take *k*_3 _value as 2.5e4.

When *z *is very small, *z *is close to *cn*. That may make organisms once generate less than one cell. To avoid this kind of error, the formula can be calibrated as:

$$I_{3}=\frac{k_{3}\cdot cn}{1+k_{3}\cdot cn/z}[\log(cn)+\log(z)]$$

The final formula to calculate the effective information of Eukaryotes is:

$$I=6ng+110x\cdot \log(6500)+\frac{2.5\times 10^{4}\cdot cn}{1+2.5\times 10^{4}\cdot cn/z}[\log(cn)+\log(z)]$$

The *x *in the formula is the size of complete transcriptome. Because the size of proteome before post-translational modifications is still unknown, we use the size of complete transcriptome instead.

The sizes of complete transcriptome come from clustered EST data. As the number of genes is quite different from the number of clustered EST, it is a problem how to use the data from EST databases. There are a few databases having EST fragments and mRNA assembled and clustered to reduce the redundancy for gene discovery, but different databases give different results. For example, the number of human UniGene clusters is about 120,000, while the number of unique sequences of human EST clusters in The Gene Index project (TGI) of Harvard University is 1,080,000 [13,14]. The difference between the two databases is supposed to be that TGI separated alternative splicing sequences and tried to produce tentative consensus, while UniGene put all the overlapping sequences together in one cluster. However only knowing this does not help match the data from the two databases. We still did not know the actual size of human complete transcriptome.

Even in one database, the data are often conflicting each other. For example, the number of human UniGene clusters is about 120,000 [15]. UniGene means unique gene, and is supposed to cluster the transcribed ESTs and mRNA into unique genes. So the number of UniGene clusters should be equal to the number of genes. However, there are only about 25,000 genes in human genome, while the number of UniGene clusters that contain only one sequence is more than 40,000. There must be errors inside. Zhang et al analyzed the results of UniGene clusters [16] and pointed out that most narrowly expressed transcripts (NETs), whose expression is confined to a few tissues, resemble intergenic sequences, and most NETs are singleton clusters containing only one EST or mRNA sequence. So those singleton clusters seem unreliable. The sequences in these clusters may come from non-coding RNA, contamination of pre-mRNA, genomic DNA, non-canonical introns or foreign sources, or simple sequencing errors.

Owing to the establishment of other specialized databases, we can resolve this problem, at least the size of the human transcriptome. The Alternative Splicing Prediction Data Base (ASPicDB) in Italy has predicted almost all human alternative-splicing transcripts and has them listed in detail [17]. They analyzed 18,193 human genes and found 319,745 transcripts, which on the whole can represent the size of complete transcriptome because their data correspond quite well with the data from TGI.

In TGI's data, 730,000 of 1,080,000 human unique consensuses are singleton. The total number of human genes is about 25,000, among which 35~60% contain alternative splicing, so the number of singleton should be about 10,000~15,000. Obviously, most sequences in the singleton are the result of errors. The real singleton sequences perhaps are already included in the tentative consensuses (TC). So we discarded all unique singletons and only count the number of TCs and obtain the result of 328,301. As ASPicDB only analyzed 18,193 genes, while human beings have 25,000 genes. If other genes do not have alternative splicing, then the size of total transcriptome should be 326,552, which is quite close to the result of TGI. It is a pity that only human data in ASPicDB is fairly comprehensive, the data of other species are quite sparse and cannot be used in the same manner.

Because TGI is not updating the data regularly and many data was released in 2006, which may be out of date, so we use UniGene data as supplement. In order to take advantages of both UniGene and TGI, we took the average value of the two databases as the complete transcriptome size *x*.

We discarded all the error prone clusters in UniGene that contain only one sequence. Having the number of the remaining UniGene clusters multiplied by possible average number of alternative splicing, we could match the two databases. For example, the number of human UniGene clusters after the treatment is 82,718. After multiplied by the possible number of alternative splicing 4, we got 330,872, which is close to the result of TGI. The UniGene result of mouse is 56,365 4 = 225,460, which is close to TGI result 210,249. We supposed the average number of alternative splicing for mammals is 4, for birds is 3, for fishes, amphibians and chordates is 2, for other animals is 1.5, for plants is 2~2.5, to make the results from the two databases as consistent as possible. We also referred to other databases.

The sizes of complete transcriptome of some eukaryotes are as follows (Table 3). The species were chosen for two reasons: first, we chose the species with higher numbers of UniGene clusters among the close species because the data are still incomplete; secondly, the species should have TGI data or other data sources. Plant species were chosen only to illustrate that this method can apply to plant.

**Table 3:** The size of transcriptome of eukaryotes

Species name	UniGene count	Alternative splicing	UniGene results	TGI results	Other sources	Transcriptome size
Homo sapiens	82,718	4	330,872	328,301	326,552 [17]	328,575
Mus musculus	56,365	4	225,460	210,249		217,855
Rattus norvegicus	40,563	4	162,252	76,570		119,411
Bos taurus	33,285	4	133,140	90,392		111,766
Sus scrofa	42,652	4	170,608	110,744		140,676
Gallus gallus	28,917	3	86,751	70,379	85,486 [31]	80,872
Xeneopus laevis	30,638	2	61,276	56,494		58,885
Xenopus tropicalis	34,428	2	68,856	69,590		69,223
Danio rerio	37,236	2	74,472	91,901		83,187
Oncorhynchus mykiss	24,527	2	49,054	40,320		44,687
Salmo salar	29,291	2	58,582	53,602		56,092
Ciona intestinalis	24,757	2	49,514	49,016		49,265
Branchiostoma floridae	13,294	2	26,588		24,020 [32]	25,304
Strongylo. Purpuratus	16,101	1.5	24,152		21,290 [33]	22,721
Aedes aegypti	17,279	1.5	25,919	25,627		25,773
Drosophila melanogaster	15,090	1.5	22,635	36,335		29,485
Lxodes scapularis	14,084	1.5	21,126	20,932		21,029
Caenorhabditis elegans	17,736	1.5	26,604	27,118		26,861
Schistosoma mansoni	9,909	1.5	14,863	19,291		17,077
Hydra magnipapillata	9,156	1.5	13,734	15,510		14,622
Zea mays	51,520	2	103,040	112,156		106,598
Triticum aestivum	32,260	2.5	80,650	93,508		87,079
Oryza sativa	34,913	2.5	87,283	82,830		85,057
Vitis vinifera	18,266	2	36,532	34,154		35,343
C. reinhardtii	8,162	2	16,324	15,761		16,043
S. pombe				5,206		5,206

In general, the results from the two databases are consistent. Some results of mammals obtained from TGI are lower than UniGene's. It's probably because some of TGI's data are too old, or because there are real differences in the average number of alternative splicing among mammals, but if so, it will be difficult to understand the huge difference between mouse and rat. At the present time, although the transcriptome data (clustered EST data) of many species are available, most of them are incomplete.

The TGI result of *Danio rerio *is too high. We cannot explain why. Perhaps those clusters contain too much gene fragments. The data of UniGene and TGI are far from perfect because they cannot correspond to the number of genes. Only if every transcript corresponds to every gene, like ASPicDB, the data can be more reliable.

**Figure 1:**
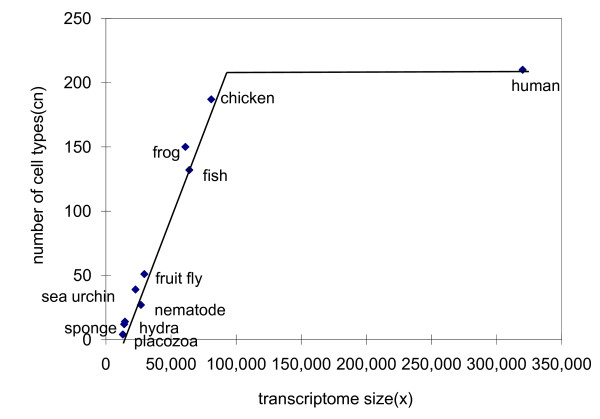
**Cell-type counts of animals plotted against the complete transcriptome size**. The number of cell types cn increases linearly with x, until reaching saturation at mammal's 210.

The data of the number of cell types of eukaryotes can be calculated. We know the number of cell types of adult human body is 210 [18]; sponges have 12 kinds of cell types [19]; the simplest multicellular organism *Trichoplax adhaerens *has 4 types of cell [20]; *C. elegans *has 27 types of cell [21]. Because the tanscriptome size of specific cells are relatively fixed, it can be anticipated that the larger the size of complete transcriptome of an organism, the more the number of cell types. Based on the data of Valentine's [22], a linear relationship between the number of cell types *cn *and the size of complete transcriptome *x *can be drawn (Fig 1). The number of cell types can be roughly calculated as:

cn=0.00273(x−11000)

There is not yet evidence if the formula apply to plants, but the *cn *of plants are calculated using the formula in this paper just to illustrate rough range of effective information of plants.

With all the data available, the effective information of eukaryotes can be calculated (Table 4). If the genomes of some organisms are not yet sequenced, the numbers of genes are estimated according to the close species (marked by * sign). Because eukaryotes also have the problem of gene overlapping, it is better to find the exact size of protein coding area to calculate *I*_1_. Sometimes the size of protein coding area is quite different from the result calculated from the number of genes. For unicellular organisms, if the transcriptome sizes *x *are not available, then *x *are the numbers of genes.

**Table 4:** The amounts of effective information of eukaryotes

Species name	x	W(g)	Gene or *I*_1_	*I *(bit)	C (bit)	*I*/C
*S. pombe *(yeast)	5,206	9.5e-11	1.4e7bits[34]	1.59e7	2.76e7[35]	58%
*S. cerevisiae *(yeast)	5,570[36]	9.5e-11	1.7e7bits[34]	1.91e7	2.42e7[37]	79%
*C. reinhardtii *(green alga)	16,043	9.5e-11	3.3e7bits[34]	3.97e7	2.4e8[38]	17%
*P. tetraurelia*(ciliate)	39,642[39]	1.0e-7	2.2e7bits[34]	4.02e7	1.44e8[34]	28%
*T. adhaerens*(placozoa)	13,000*	1.57e-10	2.65e7bits[34]	4.68e7	9.8e7[20]	48%
*H. magnipapillata*(hydra)	14,622	4.0e-4	11,648*	5.67e7	2.3e9[34]	2.5%
*S. mansoni*(trematode)	17,077	9.5e-3	3.0e7bits[34]	6.59e7	7.6e8[34]	9.7%
*C. elegans*(nematode)	26,861	3.14e-7	4.97e7bits[34]	8.71e7	2.0e8[37]	43%
*D. melanogaster*(fly)	29,485	1.96e-3	3.9e7 bits[34]	1.09e8	3.6e8[40]	29%
*L. scapularis*(tick)	21,029	1.63e-2	20,467[34]	1.05e8	4.2e9[41]	2.5%
*A. aegypti*(mosquito)	25,773	0.02	15,419[42]	1.08e8	2.7e9[42]	4.0%
*S. purpuratus*(sea urchin)	22,721	52.3	23,300[43]	1.27e8	1.6e9[43]	7.9%
*B. floridae*(amphioxus)	25,304	0.15	21,900[44]	1.27e8	1.04e9[44]	12%
*C. intestinalis*(sea squirt)	49,265	15	4.2e7bits[34]	2.20e8	3.2e8[45]	69%
*O. mykiss *(fish)	44,687	10k	25,000*	2.48e8	5.38e9[46]	4.6%
*Salmo salar*(fish)	56,092	5,500	25,000*	3.02e8	5.97e9[46]	5.1%
*Danio rerio *(fish)	83,187	0.96	6.3e7bits[34]	3.72e8	2.8e9[37]	13%
*X. laevis *(frog)	58,885	200	30,000*	3.14e8	7.02e9[46]	4.5%
*X. tropicalis*(frog)	69,223	25	28,000[37]	3.45e8	3.4e9[37]	10%
*G. gallus*(chicken)	80,872	1000	6.59e7bits[47]	4.13e8	2.0e9[37]	21%
*Bos Taurus *(cow)	111,766	1300k	22,000[37]	5.28e8	6.0e9[37]	8.8%
*R. norvegicus *(rat)	119,411	300	9.35e7bits[47]	5.09e8	5.6e9[37]	9.1%
*Sus scrofa *(pig)	140,676	100k	22,000*	5.49e8	5.4e9[48]	10%
*Mus musculus*(mouse)	217,855	25	1.0e8bits[47]	6.34^e^8	5.0e9[37]	13%
*Homo sapiens*(human)	328,575	65k	9.0e7bits[47]	8.38e8	6.4e9[37]	13%
*Vitis vinifera*(grape)	35,343	500	23,335[34]	1.89e8	9.3e8[34]	20%
*Oryza sativa*(rice)	85,057	100	6.0e7bits[34]	4.10e8	8.4e8[37]	49%
*T. aestivum*(wheat)	87,079	100	48,000*	4.89e8	3.38e10[49]	1.4%
*Zea mays*(maize)	106,598	1000	32,000[37]	4.93e8	5.6e9[37]	8.8%

After attentive observation of the *I *values, one can clearly see that the results demonstrate a definitive correlation between the amounts of effective information and the organismal phenotypic complexity defined by biological taxonomy and evolutionary theory. *C. pombe *has the lowest *I *value, while human beings have the highest. Nematode, insects, amphioxus, fish, frog, bird falls in between. The *I *values of eukaryotes range from tens of thousands to hundreds of thousands of bits, which are just one order of magnitude higher than prokaryotes'. These results are also consistent to our intuition about organismal complexity, whilst the number of genes is a poor index here. *P. tetraurelia *has quite high number of genes 39,642, but is actually a simple single cellular organism with low *I *value. So the effective information is a much better index of organismal phenotypic complexity.

## Discussion

The *I *value of *Danio rerio *(Zebra fish) is too high because the *x *is too high (higher than chicken's). The effective information of *Ciona intestinalis *accounts for 69% of the genome (almost all the non-repetitive sequence), that means the genome is quite compact and there are less junk DNA, therefore it will be easier to study the genomic information structure of this organism.

The *x *should be the number of transcripts that produce functional protein sequences. One transcript should correspond to one protein sequence, and vice versa. Every UniGene cluster should correspond to one gene and every TC in TGI should correspond to one protein sequence, and vice versa. ASPicDB uses a better algorithm because it makes all transcripts correspond with genes and proteins very well. The value of *x *is very important because the effective information is roughly proportional to *x*. The diversity of proteins before post-translational modifications can reflect the complexity of organisms.

The number of cell types was calculated with all neuron cells as one type. Some argued that neurons should be counted as different kinds of cells because they are functionally differentiated. It is difficult to count the number of neuron types at the present time. Perhaps in the future, a more objective and high throughput method can be found to count the number of neuron types. This may explain why the number of cell types reaches saturated at mammal level.

Among the alternative splicing isoforms in organism transcriptome, how many are functional is still disputable. Some alternative splicing may cause premature termination codon (PTC); and the alternative isoforms with PTC can be potentially targeted for degradation by the nonsense mediated mRNA decay (NMD) surveillance machinery. According to ASPicDB, the number of transcripts of the human genome is about 320,000, among which only 30,000 may generate PTC+ isoforms. If this part is discarded, there will be no important effect on the calculation. As PTC related data of most organisms are still unknown, this part is not taken into consideration at this time.

There is an implicit assumption in this paper: all prokaryotes have the same *k *value and so do eukaryotes. It needs to be verified for this assumption to hold. The exact values of *k *can be determined when the genomic information structures of model organisms are completely clear. For example, when the genomic information structures of *C. elegans *are completely known, the value of *k*_2 _can be completely determined. When the genomic information structures of *C. intestinalis *are known, the value of *k*_3 _can be more precisely determined.

The information contained in *I*_2 _and *I*_3 _cannot be included in the sequences of regulatory proteins. *I*_2 _is composed of regulatory sequences or regulatory factor binding sites. *I*_3 _may be composed of regulatory non-coding RNA sequences.

## Conclusions

It is clear that the effective information increases along with the increase of organismal phenotypic complexity defined by taxonomy, evolution, and intuition. The simpler the organism, the lower the *I *value. The more complex the organism, the higher the *I *value. The effective information in viruses is between 10^3^~10^5 ^bits, while in bacteria is between 10^6^~10^7 ^bits, and in eukaryotes between 10^7^~10^8 ^bits. For multicellular organisms, the effective information increases from 4.68e7 bits of placozoa to 8.38e8 bits of human beings. Worm, insects, amphioxus, fish, frog, bird falls in between. These results are consistent to other observations with the number of cell types [22], and the number of miRNA families [23]. Aburomia et al calculated the morphological complexity of 21 extant higher-order chordate groups based on the presence or absence of 479 morphological characters[24]. Their results are consistent to ours. Therefore, the effective information can be used as a quantitative measure of organismal phenotypic complexity from the simplest viruses to the most complex human beings. While limited by the incomplete data presently available, some results may not be so accurate, but the approximate range will remain true. When data become more complete and accurate, more precise calculation can be conducted.

Studies have reported increasing morphological complexity in multiple parallel lineages of the Crustacea [25]. When the phenotypic complexity of more organisms can be precisely calculated as effective information, it will be easier to study the evolution of organismal complexity.

The results of effective information of mammals are also consistent to the recently published article regarding the amount of constrained sequence in genome shared between eutherian mammals [26]. The constrained sequence means the sequence under functional constraint. The total amount of constrained sequence in rodents is estimated as 260Mb (5.2e8 bits), which is close to 5.09e8~6.34e8 bits effective information of rat and mouse. 300Mb (6.0e8 bits) of human genome is estimated under functional constraint. This is also close to 8.38e8 bits effective information of human. For fruit fly, the amount of constrained sequence is estimated as 55.5~66.2Mb (1.1 ~ 1.32e8 bits), which is also close to 1.09e8 bits effective information. Therefore, the effective information can be used as an estimate of functional fraction of genome.

## Materials and Methods

The genomic information of viruses, bacteria, and some eukaryotes can be found at GenBank. To access the GenBank, go to http://www.ncbi.nlm.nih.gov/sites/entrez?db=Genome&itool=toolbar. Input the name of the organism in the search bar and search. Then click the name again in the result page. Click the genome sequences, you can get the genome information of the organism, including number of genes, number of protein coding, percentage of coding, etc. What we need is the number of protein coding, which does not include RNA genes and pseudogenes. The size of protein coding area can be calculated as: *I*_1_=length %coding 2. The genomic information of all sequenced organisms can be obtained in this way. For the organisms with chromosome mapping, sometimes the statistic information of the chromosome sequences cannot be obtained directly. You have to click the mitochondria sequence first, and use the links in the results page to other genome sequences, to enter the statistic information pages of the chromosome sequences. The sizes of protein coding areas of some eukaryotes can be found in this manner, but some eukaryotes do not have percentage of coding data.

The value of y is calculated by bacterium volume v multiplying 16% as the weight of proteins, and divided by protein molecule average weight q.

$$\begin{array}{l} \text{y}=\text{v}\cdot 16/\text{q}\\ \text{q}=308\cdot (128-18)/6.02\text{e}23=5.628\text{e}-20 \end{array}$$

where 128 and 18 is the mean molecular weight of amino acids and water respectively, 6.02e23 is the number of molecules of one mole.

The data for *x *comes from the genome database of GenBank. The data for volumes of bacteria can be found at website: http://www.ionizers.org/Sizes-of-Bacteria.html. The volumes of bacteria can be calculated based on rod lengths and rod or coccus diameters.

Given the average cell volume v of multicellular eukaryotes as 300 μm^3^, the total cell number z of an organism is: z = w/v = (w/3) 10^10^, where w is the weight of the organism.

The data of UniGene can be found at website: http://www.ncbi.nlm.nih.gov/sites/entrez?db=UniGene. On the homepage of UniGene, you can see the number of UniGene clusters of diverse organisms. Click on the organism name and enter the page of statistic data, you can see "Final Number of Clusters" and "Histogram of cluster sizes". With the number of total sets subtracting the number of cluster sizes with only one sequence, you can obtain the number of UniGene clusters.

TGI's data can be found at website http://compbio.dfci.harvard.edu/tgi/tgipage.html. Click on the organism names, you can enter the database of that organism. There are statistical data for every organism. Only the number of TC sequences is what we need. The singleton data can be ignored.

Alternative splicing data can be found at http://t.caspur.it/ASPicDB/. There is statistic data of human alternative splicing on the homepage, including number of genes, transcripts, etc. Although there are also alternative splicing data of other species, they are not yet complete enough. You can also obtain the numbers of genes expressed in a specific tissue from this database. In the advanced search page, you can search genes with different specific tissue names in the search bar, and then you can get the number of genes. We had these numbers averaged and got about 5000. The database does not give the data of transcripts expressed in different tissues; otherwise the average size of transcriptome t can be obtained this way.

The data of average adult weights of eukaryotes are calculated based on the body sizes, which can be found or estimated from various sources.

All the calculation can be conducted by simple Perl scripts, which are available on request.

## Competing interests

The authors declare that they have no competing interests.

## Authors' contributions

YJ proposed the ideas, conducted the calculations, and wrote the manuscript. CX directed the research and have given final approval of the version to be published.

## Reviewers' comments

### Reviewer #1: Dr Lavanya Kannan (nominated by Dr. Arcady Mushegian) and Dr. Arcady Mushegian

The paper presents a method to calculate the phenotypic complexity of organisms. The phenotypic complexity of an organism is a measure of uncertainty associated with the size of the genome sequence, and is mathematically defined as the information entropy of the system. The amount of effective genomic information needed to produce a gene/protein sequences from a random sequence is at least the information entropy. The approach uses permutation and combination formulas to model the information needed to encode proteins for simple organisms like viruses, bacteria and other single celled organisms; and also extends the method to compute the information needed to produce differentiated cells and to construct spatial structures formed by the cells in higher organisms. The approach is not without interest, but several questions need to be addressed.

1. The main question is whether the complexity estimates given by the computations in this manuscript are any better than simply the number of protein-coding genes. Examining the I values for various species, from viruses to higher eukaryotes, one gets an impression that I is roughly proportional to gene numbers. Is this the case or not? If yes, what is the advantage of using I, and if no, then the relationship between the two is worth discussing in some detail.

2. In the calculation of I =Σn 6 for viruses (which is also I_1 _for all the higher organisms discussed), where Σn is the summation of the lengths of all protein sequences, the authors make the following note: I is the same as the size of the protein coding area in the DNA sequence. It would be helpful if this equality may be explained. This paragraph, as many others, suffers from simplistic explanation of biological phenomena. In p.4:\...there are no regulations of quantity of gene products. Viruses only need to produce their proteins one by one in order." - this is not true, most if not all viruses have elaborate mechanisms of, e.g., separating the expression of early and late proteins; of maintaining quite precise ratios of different protein products expressed simultaneously; etc. Many of these processes require action of virus-encoded signals and cellular proteins. But is this relevant for computing complexity? in p. 5:\In fact, mutations are not normally allowed for a real protein sequence" - not true, viruses are notorious for rapid evolution that is facilitated, in the case of RNA viruses, by a particularly high mutation rate (but again, is this information even needed for what authors are proposing?). Why does the quantity I hold for cases of overlapping genes? A simple example that exemplifies both the above facts would be beneficial for the readers.

3. In the calculation for eukaryotes: For genomes that are not sequenced, it is noted that I_1 _=6n g. It is also mentioned that in the case of overlapping genes, this quantity should be adjusted to the size of the protein-coding genes. How can this be done for the genomes that are not sequenced? Can this be elaborated?

Authors' response

The comments are insightful. We have revised the manuscript based on the review, especially the calculation of effective information of viruses.

### Reviewer #2: Dr. Chao Chen

The paper concludes that phenotypic complexity of life evolves in a single direction toward higher effective information measured by Shannon entropies. For instance, the effective information for the eukaryotes is calculated as sum of Shannon entropies corresponding to information needed (1) to encode all proteins, (2) to produce all he differentiated cells, and (3) to construct the spatial structure. For the three organisms (viruses, bacteria and eukaryotes) considered in the paper, the effective information is shown to be increasing in the order from virus to bacteria and to eukaryotes. What the authors had done is to use Sharron's information entropies to represent genotypic complexity on the three organisms but failed to demonstrate the relevance of this information to the evolution of phenotypic complexity. It should be noted that the scalability of genotypic complexity does not automatically lead to the scalability of phenotypic complexity that is not defined in the paper.

Here are my specific comments:

1. A logical framework must be constructed so that the information on genotypic complexity as measured by Shannon's information entropies can be used to support the evolution of phenotypic complexity. This framework should be equally applicable to either the entire universe of organisms or only to a single organism such as eukaryotes. The authors may first focus on one organism and then apply the methodology to a broader subgroup of organisms if not the whole universe of organisms. An interesting work on phenotypic complexity is by Lehere and Haddow [27] in which mapping of genotypic complexity to phenotypic complexity was considered.

2. In view of the comment #1 above, it is logical to consolidate all equations that are now scattering over three sections into a single section of method and/or theory. In other words, the metric on genotypic and phenotypic domains must be well defined first before any evolutionary claim could be made. Some formality on how these equations are derived should be presented. For instance, it would be more appropriate to define a1 along with a2, a3,..., acn, rather than skiping a1 and replace it by t (the averaged size of cellular proteome of differentiated cells) as shown in the section "The calculation of effective information of eukaryotes". While the calculated result will be the same, it is desirable to have some formality in presentation to avoid misunderstanding from readers. For instance, one may question the meaningfulness of the term (choosing t from x) when t is not an integer.

3. Another loose end of the manuscript is the lack of a well-defined universe from which representative samples are taken and the inference made. The scope of organisms to be inferred must be clearly defined; otherwise, the conclusion of the paper could be predetermined simply by the choice of database and the methodology. A biologist may argue that there is always an increase in complexity if one follows the simple dictum that eggs come from preexisting eggs and multicellular organism evolves from single cell animals. Questions can then be raised if viruses are therefore more primitive than rickettsiae, bacteria, fungi, algae, plants or animals. Indeed, a single cell alga is more complex than bacteria. Can gastrula be more advanced than a parasite in the gut of a termite? Can an organism like a viroid more primitive than free living algae? To prevent such issues, the domain of study and the scale of complexity must be clearly defined.

4. The manuscript needs a technical editing. Just to mention some example problems here: the sentence "Set × is the size of complete proteome.." is not clear. I believe what authors intend to say is "Let × be the size of complete proteome..". I also notice that the word "once" has been misplaced or misused in some sentences. Note that "once synthesized" means differently from "synthesized at once"

Authors' response

The comments are constructive. We have revised the manuscript based on the comments. We focus on the methodology how to calculate effective information rather than evolutionary claims in this manuscript.

### Reviewer #3: Dr. ED Rietman (nominated by Dr. Marc Vidal)

The authors are to be commended for taking on a challenging and important biological question. Their basic hypothesis is that one can use the standard information measures on DNA strings and induce similar information measures on numbers of proteins in all types of cells. The premise is that there is in increase in complexity over the course of biological evolution and that this increase in complexity comes about as a result of a reduction in entropy.

The soundness of the hypothesis will not be commented upon, because there is so much doubtful with the basic premise. Start with a simple self-replicating autocatalytic set of molecular species. If mutation-based evolution can operate on this set, there will be an increase in the complexity of the molecular species in the set. This increase in complexity is driven primarily by chemical potential and reduction in free energy. The increase in the number of new molecular species capable of participating in the reaction set results in more ways to dissipate the free energy, and thus an increase, not a decrease, in entropy [28-30].

Similarly, in a microbiological ecosystem with competing microorganisms, mutation-based evolution will increase the microorganism-based species diversity and drive up the number of ways the free energy may be dispersed. Again this results in an increase in entropy.

Besides misunderstanding fundamental thermodynamic issues, there are cellular biology errors. To support the calculations it is assumed that all proteins are produced at once. This conjecture can again be argued away, because chemical reaction networks cannot produce all molecular species at once. The chemical potential imbalance, at various points in the network, is the driving factor to produce other chemical species (Le Chatelier's principle). This same logic carries over into molecular systems biology and thus into cellular biology.

There is an insufficient review of the pertinent literature. The paper is not a review, but still a few paragraphs of review of other approaches to addressing this important question would have put this new work in perspective.

Authors' response

It is true that mutation-based evolution is an entropy increase process, but evolution may not only be mutation-based. There may be other evolutionary mechanisms. Anyway, we have deleted those parts regarding entropy in this manuscript.
